# Psychometric validation of the celiac disease-specific quality of life survey (CD-QOL) in adults with celiac disease in the United States

**DOI:** 10.1007/s11136-023-03380-7

**Published:** 2023-03-16

**Authors:** Cara Dochat, Niloofar Afari, Danielle Arigo

**Affiliations:** 1 San Diego State University/University of California San Diego Joint Doctoral Program in Clinical Psychology, 6363 Alvarado Court, San Diego, CA 92120 USA; 2 VA San Diego Healthcare System, San Diego, CA USA; 3 University of California San Diego, La Jolla, San Diego, CA USA; 4 Rowan University, Glassboro, NJ USA

**Keywords:** Celiac disease, Quality of life, Measurement, Confirmatory factor analysis

## Abstract

**Purpose:**

Celiac disease and its treatment negatively impact quality of life, indicating potential need for measurement of disease-specific quality of life domains to inform interdisciplinary intervention. The Celiac Disease Quality of Life Survey (CD-QOL) has been used in clinical research; however, its factor structure has not been confirmed and psychometric properties have not been evaluated in English-speaking adults in the U.S. Aims: (1) Confirm the factor structure of the 20-item English CD-QOL; (2) assess psychometric properties including internal consistency reliability, convergent validity, known groups validity, and incremental validity.

**Methods:**

453 adults with self-reported Celiac disease (*M*_*age*_ = 40.57; 88% female; 92% White) completed the CD-QOL and validated measures of generic health-related quality of life (SF-36), gluten-free diet adherence (CDAT), anxiety and depression symptoms (PROMIS), and physical symptoms (CSI) as part of the iCureCeliac^®^ patient-powered research network.

**Results:**

Confirmatory factor analysis found superior fit for a bifactor structure with one general factor and four group factors. Ancillary bifactor analyses suggest the CD-QOL can be considered primarily unidimensional. Total and three subscale scores demonstrated acceptable internal consistency reliability. Convergent and known groups validity were supported. The CD-QOL demonstrated some incremental validity over the SF-36.

**Conclusion:**

The English CD-QOL can be used as a measure of disease-specific quality of life among adults with Celiac disease in the U.S. Compared to generic instruments, the CD-QOL appears to better capture specific cognitive and affective aspects of living with Celiac disease. Use of a total score is recommended. Its utility as a screening and outcome measurement tool in clinical settings should be examined.

## Introduction

Celiac disease (CeD) is an autoimmune condition affecting at least 3 million people in the U.S. and 48–300 million worldwide [1, 2]. In CeD, ingestion of gluten, a protein found in wheat and some other grains, prompts an autoimmune response that causes damage to the structure and function of the small intestine. CeD often presents with aversive gastrointestinal symptoms and extraintestinal symptoms that include headache, fatigue, skin manifestations, neurologic conditions, and psychiatric conditions [3]. CeD is more prevalent in women than men worldwide [2, 4] and is diagnosed more often in non-Hispanic Whites than other racial/ethnic groups in the U.S. [5].

The only available treatment for CeD is to consume a strict, lifelong gluten-free diet (GFD), which often requires significant changes to one’s diet, increases the cost of food, and impacts functioning in multiple life domains. Individuals with CeD report high treatment burden [6] and negative impacts to quality of life [7, 8]. Lower quality of life is associated with persisting physical symptoms despite GFD adherence [9], greater depression symptoms [10], and presence of psychiatric, neurologic, and/or gastrointestinal co-morbidities [11, 12]. Lower quality of life is also cross-sectionally and longitudinally related to lower GFD adherence [7, 12, 13]. Findings suggest that increasing GFD adherence may improve quality of life, and conversely, improving quality of life may increase GFD adherence. Thus, quality of life is important to assess in addition to physical symptoms and biomarkers of CeD pathology, and may be key to ensuring the highest treatment adherence and best clinical outcomes for people with CeD.

Generic health-related quality of life measures may lack sensitivity and specificity for identifying treatment needs and capturing response to treatment [14, 15] and may not be psychometrically invariant across conditions [16]. Condition-specific quality of life measures have been developed, including the Celiac Disease Quality of Life Survey (CD-QOL) [17], Celiac Disease Assessment Questionnaire [18, 19], and Celiac Disease Questionnaire [20], among which there is some conceptual overlap (e.g., social and emotional impacts, disease concern, stigma). However, the CD-QOL is unique in that it does not assess physical symptoms, in part because participants in the development samples did not report symptoms as salient concerns, and in part due to empirical findings that quality of life in CeD is more strongly related to psychological and social functioning than symptom burden [10, 21], and changes in quality of life can occur over time despite no change in gastrointestinal symptoms [7]. Further, an estimated 21% of people with CeD are asymptomatic, and may experience negative impacts to quality of life for reasons other than symptoms [4]. Thus, a CeD-specific quality of life instrument that does not confound symptom burden may be highly appropriate for screening and outcomes measurement in clinical settings and behavioral research.

The CD-QOL was developed in the U.S. in the English language. Exploratory factor analysis found that its 20 items yielded four independent factors (“subscales”): (1) functional impact (“limitations”), (2) stigma and mood (“dysphoria”), (3) “health concerns,” and (4) perceptions of “inadequate treatment.” Additionally, developers provided initial evidence of internal consistency reliability, convergent validity, and known-groups validity. However, its four-factor structure and psychometric properties have not been evaluated in a separate U.S. sample as is best practice. Additionally, the developers and subsequent researchers have scored the CD-QOL using a total score, though support for a total score has not been demonstrated through factor analysis. Therefore, research is needed to establish the English CD-QOL as a reliable and valid measure of CeD-specific quality of life in the U.S., and to determine whether it is most appropriately scored as four subscales, a total score, or both. To address these critical gaps, the present study aimed to (1) examine the factor structure of the English CD-QOL using confirmatory factor analysis, and (2) assess psychometric properties of CD-QOL scores, including internal consistency reliability, convergent validity, known-groups validity, and incremental validity.

## Methods

### Participants and procedures

Participants were recruited to complete questionnaires as part of the iCureCeliac® patient-powered research network hosted by the Celiac Disease Foundation through the Celiac Disease Foundation newsletter or website. Questionnaires were completed at one timepoint, on a voluntary basis, between April 2019 and May 2020. All participants provided informed consent. Questionnaire participants were allowed to select from multiple diagnostic category options, including CeD, other gluten-related disorder, not diagnosed with a gluten-related disorder, and more. For the present analyses, only participants aged 18 years or older who reported a diagnosis of CeD made by biopsy, serology, or genetic testing, and their country of origin as ‘United States’ were selected.

The original dataset included *N* = 1269 participants, of whom *n* = 1152 were aged 18 or older, *n* = 1189 reported a diagnosis of CeD, and *n* = 1077 reported their country of origin as the U.S. When selecting on these inclusion criteria simultaneously, the resulting database included *n* = 913. Of those participants, *n* = 460 did not attempt the CD-QOL. When these cases were removed, the resulting database had *n* = 453, of which *n* = 23 were missing some data. This dataset of *n* = 453 was used for CFA. Of the *n* = 453 used for CFA, *n* = 138 did not complete the SF-36, PROMIS measures, CSI, and/or CDAT, leaving *n* = 315 for additional psychometric analyses.

## Measures

### Sociodemographic variables and clinical characteristics

Participants self-reported sociodemographic information and clinical characteristics (e.g., age at CeD diagnosis, method of diagnosis).

### CeD-specific quality of life

As described above, the Celiac Disease Quality of Life Survey (CD-QOL) [17] is a 20-item self-report instrument with four factor analytically derived subscales: limitations, dysphoria, health concerns, inadequate treatment. Participants are asked to rate items for concerns over the past 30 days on a 5-point scale from 1 (*not at all*) to 5 (*a great deal*). One item is reverse coded and item ratings have been summed to create a total score and subscale scores in prior studies. Higher scores indicate lower CeD-specific quality of life. Item content is available in full in the development article [17].

### Generic health-related quality of life

The RAND 36-Item Health Survey version 1.0 (SF-36) is a 36-item self-report instrument assessing eight domains of generic health-related quality of life [22]: physical functioning, social functioning, role limitations due to physical health, role limitations due to emotional problems, energy/fatigue, emotional well-being, general health, and bodily pain. Higher scores on each scale indicate better health-related quality of life. The SF-36 has demonstrated reliability and validity across multiple chronic illness populations, and has been used in CeD [20]. Internal consistency reliability of SF-36 scales in the current sample was high (Cronbach’s alpha [*α*] range = 0.83–0.92; McDonald’s omega [*ω*] range = 0.83–0.93). Participants also responded to a single item indicating whether “My health has improved since [CeD] diagnosis” from 1 (*not at all*) to 5 (*very much*).

### GFD adherence

The Celiac Dietary Adherence Test (CDAT) is a 7-item self-report measure of GFD adherence [23]. Items assess low energy, headaches, ability to follow a GFD while dining out, carefully considering consequences of one’s behavior, and perception of oneself as a failure, rated from 1 (*none of the time/strongly agree)* to 5 (*all of the time/strongly disagree)*. A sixth item assesses perception of the impact of accidental gluten exposure on health, rated from 1 (*very important*) to 5 (*not at all important*). A seventh item assesses number of intentional gluten exposures in the past four weeks, rated from 1 (*never*) to 5 (> *10*). Item ratings are summed to create a total score. Lower scores indicate greater adherence. CDAT scores were highly correlated with standardized dietician evaluation and with biomarkers of celiac disease-linked antibodies in the validation sample. Receiver operating characteristic curve analysis in the development sample showed that a CDAT score of < 13 likely indicates good adherence, scores of 13–17 likely indicate moderate adherence, and scores > 17 likely indicate poor adherence [23]. Internal consistency reliability in the current sample was low (*α* = 0.57).

Two CDAT items were examined individually as indicators of known groups validity: (1) ability to follow GFD when outside the home, and (2) intentional gluten exposure. Participants were also asked to report how many times in the past 30 days they were “inadvertently exposed to gluten” (*0, 1–2, 3–5, 6–10,* > *10*).

### Physical symptoms

The Celiac Symptom Index (CSI) is a 16-item self-report instrument assessing the extent to which respondents have been concerned with physical symptoms in the past four weeks [24]. Eleven items assess specific symptoms (e.g., gastrointestinal symptoms, low energy, headaches), rated from 1 (*none of the time*) to 5 (*all of the time*). The remaining five items assess physical health more generally, including subjective rating of CeD-specific health and general health rated from 1 (*excellent*) to 5 (*terrible*), physical pain rated from 1 (*none*) to 5 (*very much*), and comfort and relative health rated from 1 (*strongly agree*) to 5 (*strongly disagree*). Item ratings are summed to create a total score. Higher scores indicate worse symptomology and lower perceived health. Internal consistency reliability for CSI total scores in the development sample (*α* = 0.88) and current sample (*α* = 0.85) were good. Participants also responded to a single item indicating whether “I am symptomatic even though I follow a strict gluten-free diet” (*yes, no, I don’t know*).

### Anxiety and depression

The 4-item short forms of the Patient-Reported Outcomes Measurement Information System^®^ (PROMIS) anxiety and depression scales assess symptoms of anxiety and depression, respectively [25]. Respondents rate the frequency of symptoms in the past seven days on a 5-point scale from 1 (*never*) to 5 (*always*). Higher *t*-scores indicate greater anxiety and depression symptoms. PROMIS scales have strong psychometric properties [25]. Internal consistency reliability in the current sample was excellent for both anxiety (*α* = 0.90; *ω* = 0.90) and depression (*α* = 0.93; *ω*  = 0.93).

### Occupational functioning

Research suggests that adults with CeD miss significantly more work days than adults in the general population [26]. Participants reported the number of work or school days missed in the past 12 months due to illness from gluten exposure and CeD, respectively. Prior studies assessing the psychometric properties of CeD-specific quality of life instruments have similarly used single-item measures to assess limitations to daily functioning due to CeD and its treatment [14] and CeD-related quality of life [17].

## Statistical analyses

Confirmatory factor analysis (CFA) was conducted in MPlus version 8 [27] using the ‘MLR’ estimator, which provides maximum likelihood parameter estimates with standard errors robust to non-normality and is appropriate when some cases include missing data. CFA and single-item measure analyses were conducted on the total sample (*N* = 453). Additional psychometric analyses were conducted on the subsample with complete data (*n* = 315).

### Confirmatory factor analysis

CFA was used to examine the absolute fit of four models: (1) the original four-factor structure, (2) a second-order factor structure with four first-order factors and one global factor, (3) a bifactor model with one general factor and four group factors, and (4) a one-factor structure. Model fit was evaluated using the following indices: (a) Chi-square goodness-of-fit (*χ*^2^), (b) Comparative Fit Index (CFI > 0.90 acceptable, and > 0.95 desirable [28]), (c) Tucker-Lewis Index (TLI > 0.90 acceptable, and > 0.95 desirable [28]), (d) Root Mean Square Error of Approximation (RMSEA < 0.05 good fit; < 0.08 acceptable fit; < 0.10 poor fit [29, 30]) using a 90% confidence interval, and (e) Standardized Root Mean Square Residual (SRMR < 0.05 good fit, and < 0.08 acceptable fit [28]). Akaike Information Criterion (AIC) and Bayesian Information Criterion (BIC) were also used to compare models, where lower values indicate better model fit. Ancillary measures were calculated to evaluate dimensionality and model-based reliability for the bifactor model only [32]: Explained Common Variance (ECV ≥ 0.85 suggests unidimensionality [33–35]), Percent of Uncontaminated Correlations (PUC > 0.70 suggests unidimensionality when ECV > 0.70 [36]), Average Relative Parameter Bias (ARPB < 10–15% acceptable [34]), McDonald’s omega, OmegaH (> 0.50 acceptable and > 0.75 desirable [35, 37]), and H (> 0.80 desirable [38]).

### Reliability

Internal consistency reliability for CD-QOL total and three of four subscale scores was assessed using (a) Cronbach’s alpha (*α* > 0.80 good fit, and > 0.70 minimally acceptable fit [31]) and (b) McDonald’s omega, which assumes neither equivalent factor loadings nor normal distribution among scale items, and is therefore less prone to underestimation of composite reliability [39]. Because the ‘inadequate treatment’ subscale has only two items, alpha may underestimate their true relationship, and a Pearson correlation coefficient was calculated instead.

### Validity

Convergent validity was assessed by computing Spearman’s rho correlation coefficients for CD-QOL total and subscale scores and scores on the SF-36 scales, CDAT (GFD adherence), PROMIS anxiety and depression scales, occupational functioning items, and CSI (physical symptoms). Coefficients *r* = 0.00–0.39 were considered small, *r* = 0.40–0.69 were considered moderate, and *r* = 0.70–1.00| were considered large. Because CD-QOL items assess social limitations, emotional concerns, and cognitive concerns rather than physical aspects, we hypothesized moderate negative correlations between CD-QOL total and SF-36 social functioning, emotional well-being, and general health subscale scores, and small negative correlations between CD-QOL total and SF-36 physical functioning, role limitations due to physical and emotional problems, energy/fatigue, and bodily pain, such that worse CeD-specific quality of life would be related to worse generic quality of life.

We expected a moderate positive correlation between CD-QOL total and CDAT total, such that worse quality of life would be related to lower GFD adherence. We hypothesized moderate positive correlations between CD-QOL total and PROMIS scale scores, such that worse quality of life would be related to greater anxiety and depression symptoms. We also hypothesized moderate positive correlations between CD-QOL total and occupational functioning, such that worse quality of life would be related to more days missed (i.e., worse functioning). We also expected CD-QOL dysphoria subscale scores to be more strongly related to measures of mental health (PROMIS anxiety and depression, SF-36 emotional well-being) than other CD-QOL subscales. Because prior research suggests that the relationship between CeD-specific quality of life and physical symptoms may be limited, we expected a small, positive correlation between CD-QOL and CSI scores, with worse quality of life related to greater symptom burden.

Known groups validity was assessed by grouping participants according to established cut-off scores for GFD adherence on the CDAT and examining mean group differences in CD-QOL total score using analysis of variance and planned pairwise comparisons with Bonferroni corrections. We expected significantly greater mean CD-QOL scores (i.e., worse quality of life) among those with poor GFD adherence compared to those with good GFD adherence. Known groups validity was further assessed using independent samples *t*-tests to examine group differences in mean CD-QOL total score between participants (a) reporting *any* inadvertent gluten exposure and those reporting none, (b) reporting *any* intentional gluten exposure and those reporting none, and (c) endorsing an ability to follow a GFD when dining outside the home compared to those reporting inability. In each comparison we hypothesized significantly greater CD-QOL scores among the less adherent groups. Finally, mean CD-QOL total scores were compared between participants (d) endorsing persisting symptoms despite GFD adherence and those not endorsing, and (e) reporting significantly improved health since CeD diagnosis (*very much, quite a bit*) and those who did not (*no, a little, somewhat*). We hypothesized significantly higher CD-QOL scores among those reporting persisting symptoms and little to no improvement in health.

Incremental validity of the CD-QOL for predicting concurrent GFD adherence (CDAT scores) over and above a generic health-related quality of life measure (SF-36) was examined using hierarchical linear regression. Select SF-36 scales were entered in step 1 and CD-QOL total score was entered in step 2. Three SF-36 scales were used given their conceptual overlap with domains assessed by the CD-QOL: emotional well-being, social functioning, and general health. Models with and without CD-QOL total score were compared to determine change in total variance explained (*R*^*2*^).

## Results

### Sociodemographic and clinical characteristics

Sample characteristics are shown in Table 1. Most participants identified as women and White, and ages ranged from 18 to 83 years (*M* = 41, *SD* = 15). Age at CeD diagnosis ranged from two to 77 years (*M* = 35, *SD* = 15) and years since diagnosis ranged from zero to 78 (*M* = 6, *SD* = 8). Most participants reported diagnosis of CeD via intestinal biopsy (82%) followed by serology (16%) and genetic testing (2%). There were no differences in sociodemographic or clinical characteristics between the total sample used for CFA and the subsample used for other psychometric analyses (*p*s > 0.05).

**Table 1:** Sociodemographic Variables, Clinical Characteristics, and Mean Questionnaire Scores for Total Sample used in CFA (*N* = 453) and Subsample with Complete Data (*n* = 315)

Measure	Total (*N* = 453)	Subsample (*n* = 315)
Sociodemographic and clinical characteristics		
Age, *M* (*SD*)	40.57 (14.84)	40.94 (15.15)
Female	87.6%	87.6%
Race/Ethnicity		
White	92.3%	91.7%
Hispanic/Latino	3.5%	3.5%
American Indian/Alaskan Native	2.2%	2.5%
Black	0.7%	1.0%
Asian	0.2%	0.3%
Native Hawaiian/Pacific Islander	0.2%	0.0%
Other	0.4%	0.6%
Household Income		
Less than $50,000	23.7%	23.3%
$50,000-$100,000	36.8%	37.0%
$100,000-$200,000	29.8%	30.0%
$200,000 or more	9.6%	9.7%
Education		
High School Diploma	5.7%	5.1%
Vocational, Trade, or Associate’s	13.9%	14.1%
Bachelor’s or some college	54.0%	54.4%
Professional, Master’s, or Doctorate	26.5%	26.5%
Age at diagnosis, *M* (*SD*)	34.65 (15.00)	34.94 (15.05)
Years since diagnosis, *M* (*SD*)	5.94 (7.81)	5.93 (7.49)
Diagnostic method		
Biopsy (small bowel/intestine)	82.3%	83.8%
Serology/blood test	15.9%	14.6%
Other	1.8%	1.6%
Diagnostic reason		
Symptomatic	76.2%	76.8%
Other	23.8%	23.2%
Missed school or work days, *M* (*SD*)		
Due to illness from gluten exposure	12.25 (42.83)	13.00 (46.32)
Due to Celiac Disease	13.74 (46.00)	13.13 (45.02)
Symptomatic despite gluten-free diet adherence		
Yes	51.1%	50.7%
No	31.4%	32.6%
I do not know	17.4%	16.8%

Measures	*M* (*SD*)	*M* (*SD*)
CD-QOL Total	63.07 (16.17)	62.40 (16.17)
CD-QOL Limitations	29.77 (8.27)	29.74 (8.29)
CD-QOL Dysphoria	9.45 (4.09)	9.25 (4.04)
CD-QOL Health Concerns	17.10 (4.82)	16.76 (4.87)
CD-QOL Inadequate Treatment	6.75 (2.11)	6.66 (2.06)
SF-36 Physical Functioning		81.37 (22.55)
SF-36 Role Limitations – Physical health		55.63 (42.86)
SF-36 Role Limitations – Emotional problems		56.19 (42.56)
SF-36 Energy/Fatigue		39.83 (24.06)
SF-36 Emotional Wellbeing		63.86 (19.84)
SF-36 Social Functioning		70.79 (26.07)
SF-36 Bodily Pain		61.24 (24.99)
SF-36 General Health		51.14 (23.88)
CDAT total		13.33 (3.59)
CSI total		39.85 (9.90)
PROMIS Anxiety		54.40 (9.68)
PROMIS Depression		51.97 (9.54)

All values are raw scores except for PROMIS measures which are *t*-scores. Blank spaces indicate incomplete data for *N* = 453

*M *mean, *SD* standard deviation, *CD-QOL* Celiac Disease Quality of Life Survey, *CDAT* Celiac Dietary Adherence Test, *CSI* Celiac Symptom Index, *PROMIS* Patient-Reported Outcomes Measurement Information System^®^

### Confirmatory factor analysis

#### Model 1

Four latent variables (limitations, dysphoria, health concerns, inadequate treatment) were indicated by nine, four, five, and two items, respectively. Inter-factor correlations were specified. As shown in Table 2, the model did not fit well statistically (*χ*^2^ [164, *N* = 453] = 543.085, *p* < 0.001) and two descriptive fit indices showed borderline acceptable fit (TLI = 0.890; RMSEA = 0.071). However, the non-parsimony adjusted descriptive fit indices showed acceptable model fit (CFI = 0.905, SRMR = 0.054). Given that the Chi-square goodness-of-fit test is sensitive to sample size and is often significant for large samples, and the non-parsimony descriptive fit indices yielded desirable values, model evaluation proceeded [40]. All standardized factor loadings were large and statistically significant (*p* < 0.001): limitations (range: 0.437–0.818), dysphoria (range: 0.499–0.876), health concerns (range: 0.478–0.870), inadequate treatment (range: 0.572–0.683). Standardized inter-factor correlations were also large and statistically significant (*p* < 0.001), suggesting that these four latent factors are strongly related but not redundant (*r*s = 0.601–0.756). Inter-subscale correlations ranged from *r* = 0.42– 0.64. Model findings were replicated in the subsample with complete data on all measures (*n* = 315).

**Table 2:** Goodness-of-fit Statistics for Comparative Confirmatory Factor Analytic Models (*N* = 453)

Models	Chi-square	Df	CFI	TLI	RMSEA [90% CI]	SRMR	AIC	BIC
1. 4 factors	543.085***	164	0.905	0.890	0.071 [0.065, 0.078]	0.054	26027.293	26298.942
2. 2nd order	546.308***	166	0.905	0.891	0.071 [0.065, 0.078]	0.054	26027.086	26290.503
3. Bifactor	399.078***	150	0.938	0.921	0.061 [0.053, 0.068]	0.040	25892.013	26221.284
4. 1 factor	1069.055***	170	0.775	0.748	0.108 [0.102, 0.114]	0.068	26594.820	26841.774

All models tested using maximum likelihood estimation with robust standard errors

*Df* degrees of freedom, *CFI* Comparative Fit Index, *TLI* Tucker–Lewis Index, *RMSEA* Root Mean Square Error of Approximation, *CI* Confidence Interval, *SRMR* Standardized Root Mean-Square Residual, *AIC* Akaike Information Criterion, *BIC*   Bayesian Information Criteria

^***^p < .001

#### Model 2

**Fig. 1:**
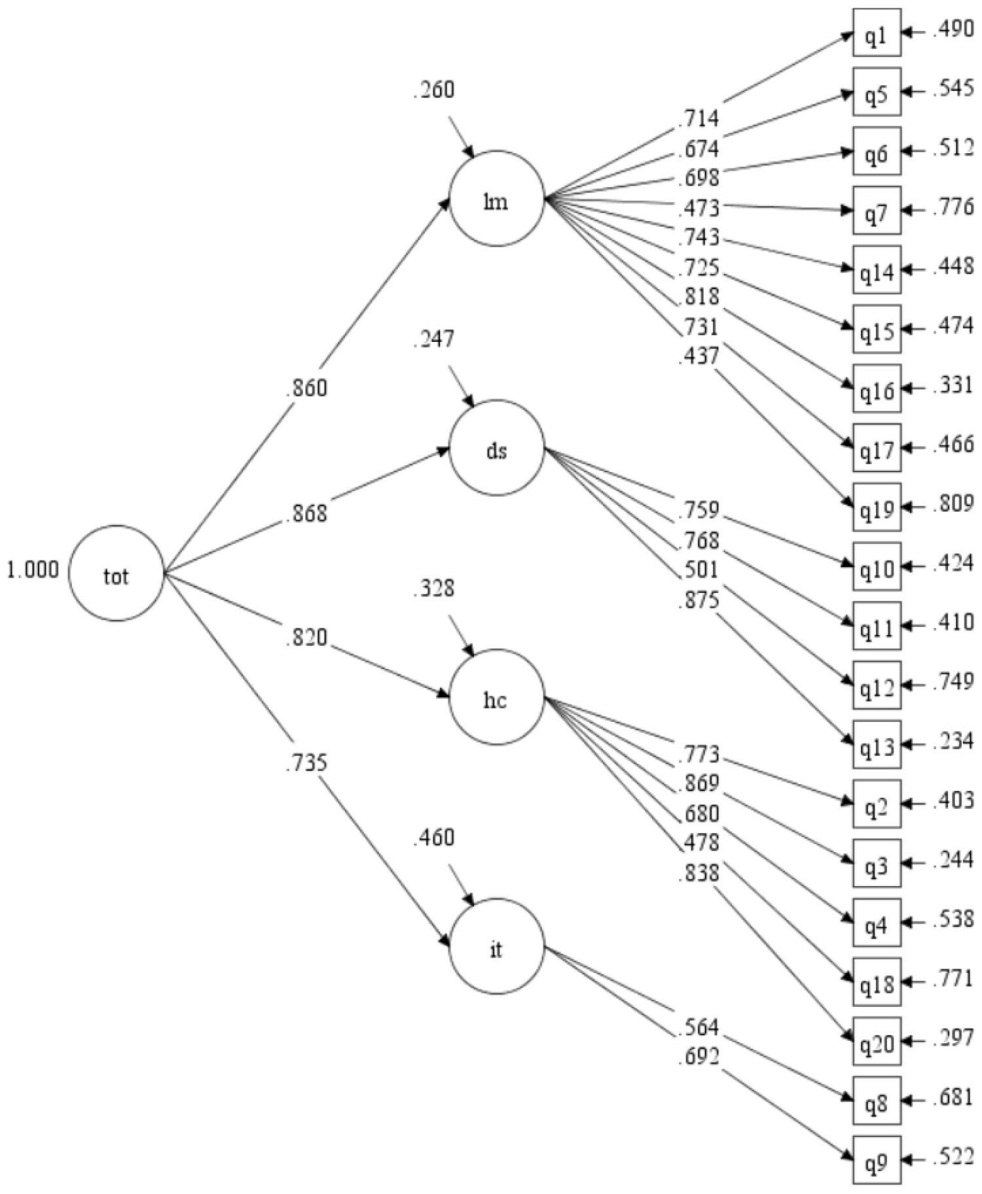
Second-Order Model Confirmatory Factor Analysis (Model 2)*.* Standardized path coefficients are shown. All paths are statistically significant (*p* < 0.001). lm = limitations; ds = dysphoria; hc = health concerns; it = inadequate treatment; tot = overall CeD-related quality of life. Brief item descriptors: q1 = Limited, q5 = Social Stigma, q6 = Limited Meals, q7 = Special Foods, q14 = Socializing, q15 = Travel, q16 = Normal Life, q17 = Contamination Fear, q19 = Think about Food, q10 = Depressed, q11 = Frightened, q12 = Lack of Information, q13 = Overwhelmed, q2 = Suffer, q3 = Other Health Problems, q4 = Cancer Risk, q18 = Family Risk, q20 = Long Term Health, q8 = Diet Sufficiency, q9 = Treatment Choices

A second-order factor structure including four first-order factors identical to those in model 1 and a single second-order factor (representing a total score) was specified. As shown in Table 2, model 2 did not fit well statistically (*χ*^2^ [166, *N* = 453] = 546.308, *p* < 0.001) and two descriptive fit indices showed borderline acceptable fit (TLI = 0.891; RMSEA = 0.071). However, the non-parsimony adjusted descriptive fit indices showed acceptable model fit (CFI = 0.905, SRMR = 0.054) and model evaluation proceeded. As shown in Fig. 1, all standardized item to first-order factor loadings were large and statistically significant (*p* < 0.001): limitations (range: 0.437–0.818), dysphoria (range: 0.501–0.875), health concerns (range: 0.478–0.869), inadequate treatment (range: 0.564–0.692). Standardized loadings between the first-order factors and second-order factor were large and statistically significant (range: 0.735–0.868). Model findings were replicated in the subsample with complete data (*n* = 315). Model 2 had lower AIC and BIC values than model 1, suggesting a better fit for model 2.

#### Model 3

**Fig. 2:**
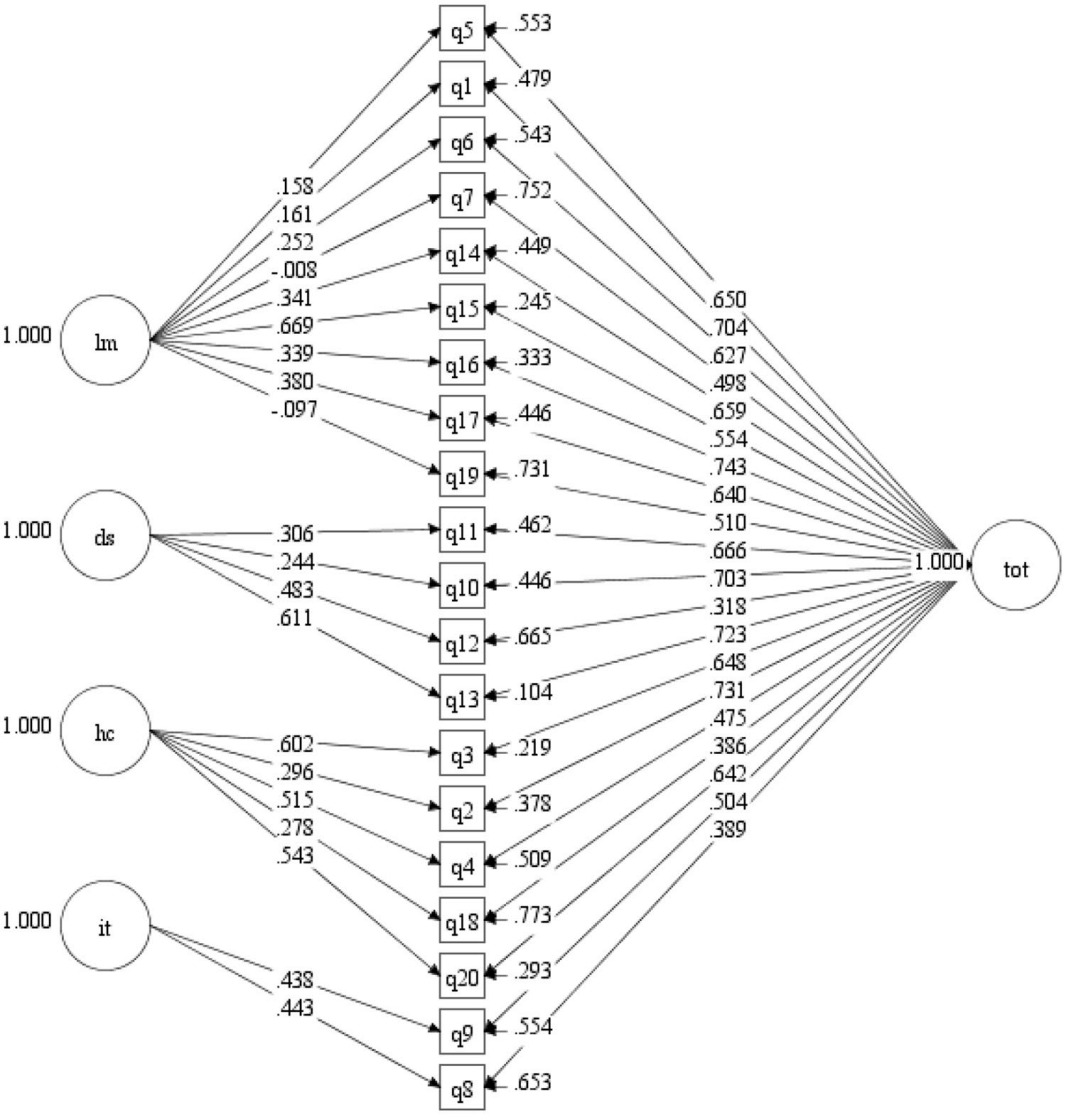
Bifactor Model Confirmatory Factor Analysis (Model 3). Standardized path coefficients are shown. All paths to tot, ds, hc, and it are statistically significant (*p* < 0.001). All paths to lm are statistically significant (*p* < 0.05) except q5 (*p* = 0.06), q7 (*p* = 0.92), and q19 (*p* =0 .10). lm = limitations; ds = dysphoria; hc = health concerns; it = inadequate treatment; tot = general CeD-related quality of life. Brief item descriptors: q1 = Limited, q5 = Social Stigma, q6 = Limited Meals, q7 = Special Foods, q14 = Socializing, q15 = Travel, q16 = Normal Life, q17 = Contamination Fear, q19 = Think about Food, q10 = Depressed, q11 = Frightened, q12 = Lack of Information, q13 = Overwhelmed, q2 = Suffer, q3 = Other Health Problems, q4 = Cancer Risk, q18 = Family Risk, q20 = Long Term Health, q8 = Diet Sufficiency, q9 = Treatment Choices

A bifactor structure including one general and four orthogonal group factors was examined. As shown in Table 2, model 3 did not fit well statistically (*χ*^2^ [150, *N* = 453] = 399.078, *p* < 0.001). However, all descriptive fit indices showed acceptable or better fit (TLI = 0.921; RMSEA = 0.061; CFI = 0.938, SRMR = 0.040), thus model evaluation proceeded. As shown in Fig. 2, all standardized item loadings on the general factor were large and statistically significant (range: 0.475–0.743; *p* < 0.001), except for items 8, 12, and 18, which showed smaller but nevertheless significant loadings (range: 0.318–0.389; *p* < 0.001). Standardized item loadings on three of the four group factors ranged in size and were all statistically significant (*p* < 0.001): dysphoria (range: 0.244–0.611), health concerns (range: 0.278–0.602), inadequate treatment (range: 0.390–0.498). Standardized item loadings on the largest group factor (limitations) ranged widely (range: − 0.097–0.669) and only six of nine item loadings were statistically significant (*p* < 0.05). Compared to model 2, model 3 had lower AIC and BIC values and better descriptive fit than model 2.

Standardized item loadings on the general factor were comparable to or greater than loadings on the group factors with two exceptions (items 12 and 20). Ancillary bifactor analyses suggested that the CD-QOL can be considered primarily unidimensional despite the presence of some multidimensionality (ECV = 0.694, PUC = 0.721, ARPB = 0.219). Analyses provided support for model-based reliability of the general factor (omega = 0.941; omegaH = 0.858; H = 0.927). Support for model-based reliability of specific factors was mixed: limitations (omegaS = 0.876; omegaHS = 0.126; H = 0.578), dysphoria (omegaS = 0.835; omegaHS = 0.265; H = 0.516), health concerns (omegaS = 0.856; omegaHS = 0.324; H = 0.324), inadequate treatment (omegaS = 0.694; omegaHS = 0.259; H = 0.259). ECV for group factors ranged from 0.223 to 0.390.

#### Model 4

For completeness, a single-factor structure was also tested. As shown in Table 2, the *p*-value for the Chi-square goodness-of-fit test was statistically significant, and values on three of four descriptive fit indices failed to meet threshold for acceptable fit. Model evaluation did not proceed.

### Internal consistency reliability

Internal consistency reliability of CD-QOL total (*α* = 0.92; *ω* = 0.92), limitations subscale (*α* = 0.88; *ω* = 0.88), dysphoria subscale (*α* = 0.81; *ω* = 0.83), and health concerns subscale (*α* = 0.84; *ω* = 0.85) scores was good. Correlation between the two items in the inadequate treatment subscale was small but close to moderate (*r* = 0.39, *p* < 0.001).

### Convergent validity

As shown in Table 3, correlations between CD-QOL total and SF-36 social functioning, emotional well-being, and general health and scores were moderate and correlations between CD-QOL total and SF-36 physical functioning, role limitations, and bodily pain were small, as expected. The correlation between CD-QOL total and SF-36 energy/fatigue subscale was slightly larger than expected. The correlation between CD-QOL total and CDAT total was slightly smaller than expected.

**Table 3:** Correlations Between CD-QOL Total, Subscale Scores, and Related Construct Scores (*n* = 315)

Construct Measure	CD-QOL Total	CD-QOL Limitations	CD-QOL Dysphoria	CD-QOL Health Concerns	CD-QOL Inadequate Treatment
Generic health-related QoL					
SF-36 PF	−0.26**	−0.20**	−0.27**	−0.22**	−0.21**
SF-36 RP	−0.35**	−0.28**	−0.32**	−0.31**	−0.29**
SF-36 RE	−0.34**	−0.29**	−0.41**	−0.23**	−0.18**
SF-36 EF	−0.43**	−0.36**	−0.38**	−0.38**	−0.29**
SF-36 EW	−0.49**	−0.43**	−0.54**	−0.36**	−0.24**
SF-36 SF	−0.48**	−0.43**	−0.49**	−0.37**	−0.24**
SF-36 BP	−0.36**	−0.27**	−0.34**	−0.31**	−0.32**
SF-36 GH	−0.50**	−0.39**	−0.46**	−0.45**	−0.35**
Gluten-free diet adherence					
CDAT total	0.38**	0.31**	0.45**	0.29**	0.28**
Anxiety					
PROMIS Anxiety	0.50**	0.41**	0.55**	0.44**	0.19**
Depression					
PROMIS Depression	0.49**	0.40**	0.54**	0.40**	0.25**
Occupational functioning					
Missed school/work days due to illness from gluten exposure	0.34**	0.26**	0.31**	0.30**	0.18**
Missed school/work days due to Celiac Disease	0.37**	0.29**	0.36**	0.31**	0.20**
Celiac disease-related symptoms					
CSI total	0.08	0.08	0.05	0.07	−0.02

Values shown are Spearman’s rho coefficients. Moderate to large coefficients are bolded

*CD-QOL* Celiac Disease Quality of Life Survey, *PF* Physical functioning, *RP* Role limitations due to physical health, *RE* Role limitations due to emotional problems, *EF* Energy/fatigue, *EW* Emotional well-being, *SF* Social functioning, *BP* Bodily pain, *GH* General health, *CDAT* Celiac Dietary Adherence Test, *PROMIS* Patient-Reported Outcomes Measurement Information System^®^, *CSI* Celiac Symptom Index

For occupational functioning items, *n* = 344

**p < .001

Also as expected, CD-QOL total and three subscales were moderately correlated with PROMIS anxiety and depression scores, and the ‘dysphoria’ subscale was more strongly correlated with measures of mental health than other CD-QOL subscales. The correlation between CD-QOL total and occupational functioning was smaller than expected. Finally, CD-QOL total and CSI scores were minimally correlated, as predicted.

### Known groups validity

As shown in Table 4, there were significant differences between CDAT adherence groups in mean CD-QOL total score, *F*(2, 311) = 20.24, *p* < 0.001. Planned pairwise comparisons revealed significant differences between good and moderate adherence groups (*p* < 0.001), good and poor adherence groups (*p* < 0.001), and moderate and poor adherence groups (*p* = 0.03) in the expected directions. Similarly, individuals who reported no inadvertent gluten exposure, no intentional gluten consumption, and ability to adhere to a GFD when dining outside the home reported significantly lower (better) CD-QOL scores than did groups with worse adherence. Those who endorsed persisting symptoms despite GFD adherence reported higher CD-QOL scores and those who reported greater improvements to health since diagnosis, as expected.

**Table 4:** Known Groups Validity and Incremental Validity Results for CD-QOL Total Score

Known groups validity	*N*	*M* (*SD*)	*F*/*t*	*p*
CDAT total			20.24	** < 0.001**
Poor adherence	39	72.28 (15.93)		
Moderate adherence	142	65.26 (15.16)		
Good adherence	133	56.63 (15.10)		
Inadvertent gluten exposure (past 30 days)			−4.76	** < 0.001**
None	89	56.67 (16.85)		
Any	251	65.64 (14.70)		
Intentional gluten consumption (past 4 weeks)			−2.44	**0.02**
Never	388	62.35 (16.04)		
Any	62	67.70 (15.91)		
Able to follow GFD outside home			−7.00	** < 0.001**
Agree	350	60.11 (15.46)		
Disagree	78	73.47 (14.21)		
Symptomatic despite GFD adherence			7.61	** < 0.001**
No	137	55.85 (16.17)		
Yes	223	68.45 (14.66)		
Health has improved since diagnosis			−4.99	** < 0.001**
Very much/Quite a bit	243	59.75 (15.60)		
No/A little/Somewhat	201	67.24 (15.89)		

Incremental validity	*B(SE*)	*F/t* (*p*)	*R*^2^	∆*R*^2^
Outcome: CDAT total				
Step 1		48.31 (< 0.001)	0.318	
Constant	19.21 (0.58)	33.4 (< .001)		
SF-36 EW	−0.02 (0.01)	−1.46 (.15)		
SF-36 SF	−0.03 (0.01)	−3.18 (**0.002**)		
SF-36 GH	−0.05 (0.01)	−5.16 (**< 0.001**)		
Step 2		25.29 (< 0.001)	0.326	0.008
Constant	16.71 (1.27)	13.20 (< 0.001)		
SF-36 EW	−0.003 (0.01)	−1.07 (0.29)		
SF-36 SF	−0.03 (0.01)	−2.95 (**0.003**)		
SF-36 GH	−0.04 (0.01)	−4.52 (**<0 .001**)		
CD-QOL Total	0.03 (0.01)	1.95 (**0.05**)		

Values ≤.05 are shown in bold

*M* mean, *SD* standard deviation, *B* unstandardized regression coefficient, *SE* standard error, *CDAT* Celiac Dietary Adherence Test, *CD-QOL* Celiac Disease Quality of Life Survey, *EW* Emotional well-being; *SF* Social functioning, *GH* General health

### Incremental validity

Step 1 of the regression model was statistically significant and explained 32% of the variance in CDAT total scores, *F*(3, 311) = 48.31, *p* < 0.001 (Table 4). When CD-QOL total was added in step 2, the omnibus model remained significant, *F*(4, 310) = 37.50, *p* < 0.001, and there was a 1% increase in variance explained. CD-QOL total and SF-36 social functioning and general health were significant predictors of CDAT score.

## Discussion

The aims of the current study were to confirm the factor structure and examine the psychometric properties of the English language CD-QOL among adults with CeD in the U.S. Previous work using exploratory factor analysis identified a four-factor solution. We extended prior work by examining a second-order hierarchical structure and a bifactor structure to address whether the measure can be appropriately scored with a raw summed total score. Of the various models tested, the bifactor model showed superior model fit. Ancillary bifactor analyses suggested that the CD-QOL can be considered a primarily unidimensional instrument, assessing a general latent factor of CeD-specific quality of life, and that the total score is more reliable than the subscale scores. Though ancillary analyses confirmed some multidimensionality of the instrument and subscale reliability indices were mixed, the subscale scores, especially for limitations, do not appear to narrowly measure the proposed specific factors and do not provide substantial interpretive value beyond what is provided by the total score. It is therefore recommended that researchers and clinicians in the U.S. using the English CD-QOL consult the total score rather than subscale scores to assess CeD-specific quality of life. Bifactor results suggested that ‘limitations’ is the least reliable and robust subscale. Item loadings to that factor were generally weaker or non-existent compared to loadings on the general factor, a situation known as “factor collapse” [41]. This finding suggests that themes among these items may best characterize the general factor, such as social stigma, social exclusion, and fear about or preoccupation with food.

In terms of the psychometric properties of the CD-QOL total score and subscale scores, the total score and three subscale scores demonstrated good to acceptable internal consistency reliability. The two items in the inadequate treatment subscale were moderately correlated and evidenced good factor loadings; however, additional items may be needed to better operationalize this subscale. Convergent and known groups validity were supported. The pattern of relationships between the CD-QOL and SF-36 suggest that the CD-QOL assesses specific aspects of quality of life that it purports to measure, and these constructs are related to but not redundant with generic health-related quality of life constructs. Incremental validity findings suggest that researchers and clinicians might choose to use either the generic SF-36 or CD-QOL to assess quality of life in adults with CeD. Selecting one or both measures may depend on the purpose [42–44]. The CD-QOL assesses aspects of functioning and well-being that may not be captured by generic measures and could be important indicators of treatment needs, such as CeD-specific social concerns, food concerns, health concerns, and affect impairments, which can be targeted with behavioral interventions. The CD-QOL is notably shorter than the SF-36, which may reduce burden on both administrators and respondents.

The present study addressed an important gap in the literature. However, our findings may not generalize to all adults with CeD in the U.S. Participants in the present study were self-selected and represent a population with access to the internet, knowledge of how to find relevant health information, willingness to be part of the research community, and capacity to complete online questionnaires. Additionally, because the iCureCeliac database did not inquire about current location, we have assumed that participants who identified their country of origin as the U.S. were living in the U.S. at the time of survey completion, which we were unable to verify. The original CD-QOL items were developed and refined using feedback from mostly White, mostly female patient groups, and as such, item wording or response options may not represent the construct adequately in other groups. Most participants in the current study also identified as female and White, which generally reflects characteristics of the U.S. CeD patient population [5, 45, 46], but may limit generalizability to other patient groups [47].

Researchers should seek to validate CD-QOL scores among individuals of more diverse gender identifications, socioeconomic resources, and racial and ethnic backgrounds. Cross-cultural research with translated versions of the CD-QOL has identified alternative factor structures and subscale composition [14, 48–50], suggesting that the presently supported factor structure may not generalize to other countries and cultures and should be evaluated carefully. Further, samples in the original validation studies and the present study had been diagnosed for an average of nine and six years, were mostly diagnosed as adults, and reported relatively high GFD adherence. The validity and utility of the CD-QOL among newly diagnosed individuals or those expressly struggling with GFD adherence should be assessed.

Another possible limitation of the current study is use of self-report measurement, including for CeD diagnostic status. Individuals were invited to participate in the iCureCeliac^®^ registry if they had a “gluten-related condition,” which includes but is not limited to CeD. To reduce demand characteristics to report a diagnosis of CeD if no diagnosis had been made, participants were invited to select from multiple diagnostic category options (e.g., CeD, other gluten-related disorder, not diagnosed with a gluten-related disorder), and were invited to participate in the registry regardless of their response. Only participants who reported a diagnosis of CeD made by biopsy, serology, or genetic testing were included in the present analyses. Notably, the genotype HLA-DQ2 or HLA-DQ8 is a necessary but insufficient condition for diagnosis of CeD. Therefore, our inclusion of *n* = 8 participants (< 2%) who reported a diagnosis of CeD by genetic testing introduces the possibility that those individuals do not meet criteria for CeD diagnosis, which may impact findings. Finally, our analyses were confined by the cross-sectional nature of the data. Future studies should capture longitudinal data to examine the CD-QOL’s test–retest reliability and sensitivity to change, which will provide information about its utility for screening and outcomes assessment purposes.

## Conclusion

The CD-QOL is a reliable and valid measure of CeD-specific quality of life. It assesses important cognitive, affective, and behavioral aspects of living with CeD that may negatively impact GFD adherence and mental health. These aspects represent appropriate targets for interdisciplinary treatment to improve health outcomes. Additional research is needed to specifically evaluate the utility of the CD-QOL as a screening tool and outcomes measure in health services research and routine clinical care.

## Data Availability

The data that support the findings of this study are available from Celiac Disease Foundation but restrictions apply to the availability of these data, which were used under license for the current study, and so are not publicly available.
